# Transient expressions of synthetic biology in plants^☆^

**DOI:** 10.1016/j.pbi.2014.02.003

**Published:** 2014-06

**Authors:** Frank Sainsbury, George P Lomonossoff

**Affiliations:** 1 The University of Queensland, Australian Institute for Bioengineering and Nanotechnology, Centre for Biomolecular Engineering, St Lucia, QLD 4072, Australia; 2 Department of Biological Chemistry, John Innes Centre, Norwich Research Park, Norwich NR4 7UH, United Kingdom

## Abstract

Recent developments in transient expression methods have enabled the efficient delivery and expression of multiple genes within the same plant cell over a timescale of days. In some cases, the vectors deployed can be fine-tuned to allow differential expression of the various genes. This has opened the way to the deployment of transient expression for such applications as the production of macromolecular complexes and the analysis and manipulation of metabolic pathways. The ability to observe the effect of gene expression in a matter of days means that transient expression is becoming the method of choice for many plant-based synthetic biology applications.

## Highlights

• Transient expression is an effective method for the co-expression of proteins.
• A number of vectors are available to facilitate this use.
• They can be used to produce complex macromolecules and analyse metabolic pathways.
• Transient expression is likely to become a method of choice for plant synthetic biology.

**Current Opinion in Plant Biology** 2014, **19**:1–7

This review comes from a themed issue on **Physiology and metabolism**

Edited by **Sarah E O’Connor** and **Thomas P Brutnell**

For a complete overview see the Issue and the Editorial

Available online 12th March 2014

1369-5266/$ – see front matter, © 2014 The Authors. Published by Elsevier Ltd. All rights reserved.

**http://dx.doi.org/10.1016/j.pbi.2014.02.003**

## Introduction

In recent years, the development and application of transient expression systems has led to significant gains in our capacity to apply the principles of synthetic biology to plants. Traditionally, modification of plants for recombinant protein expression has relied on the generation of stable transgenic lines, which is both time-consuming and resource-intensive. Transient transformation of plant tissues, by contrast, is very rapid, generating recombinant proteins or the products of their activity within days, and can now be scaled up to commercially relevant production levels. Through significant improvements in expression yields, the transient approach has become an attractive manufacturing system and has resulted in considerable progress being made in the use of plants in synthetic biology.

A number of reviews that cover the use of transient expression systems for the production of foreign proteins are available [1–3]. In the present commentary we will extend this concept to advances in synthetic biology, defined here as the construction of entities not found in nature. To introduce this, we briefly describe the development of the tools and materials that are enabling the exploration of the potential of plant-based synthetic biology for the assembly of complex heteromultimers and the exploitation of the rich resource of secondary metabolites found in plants. Although developments in transient expression resulting from deeper understanding of underlying fundamental processes have enabled advanced synthetic biology in plants, our experience is that the transient synthetic reconstruction of biological systems has also revealed the workings of otherwise intractable processes.

## The tools

### Transient expression

Taking advantage of the well established ability of *Agrobacteria tumefaciens* to transfer a defined segment of DNA (T-DNA) to the plant nucleus [4], the molecular genetic modification of plants is most often performed using binary vector systems that separate the T-DNA from the *trans*-acting virulence proteins that effect the transfer [5]. This allows for the manipulation of broad host-range plasmids of manageable size through routine molecular biology techniques as well as disarming the parent tumour-inducing plasmid. Transient expression of heterologous proteins in intact leaves was demonstrated by infiltration of suspensions of *A. tumefaciens* harbouring a binary vector into leaf interstitial spaces [6]. This ground-breaking study established ‘agroinfiltration’ as a rapid recombinant protein expression technique that is inherently flexible and scalable. Furthermore, since the *Agrobacterium* strains are disarmed and only non-reproductive tissue is modified, the process does not modify the germ line of the target plants and the process is thus considered to be environmentally benign.

### Regulatory elements

**Figure 1:**
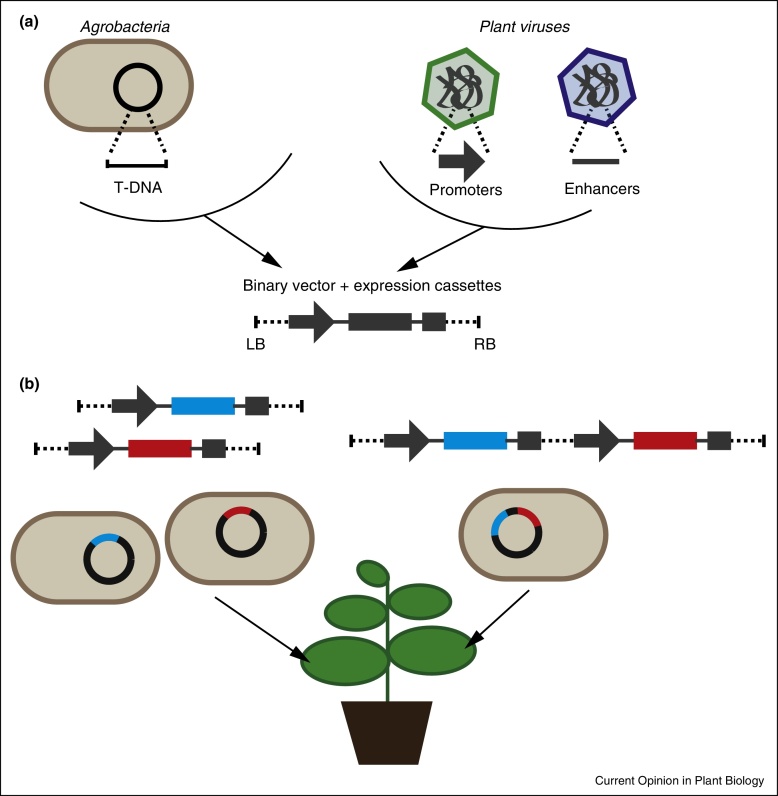
Development of transient expression systems for use in plant-based synthetic biology. **(a)** Disarming and reprogramming of the Agrobacteria tumour-inducing plasmid was combined with plant viral regulatory sequences to give binary vector systems harbouring high-yielding expression cassettes. **(b)** Co-expression may be achieved via co-infiltration of multiple Agrobacteria cultures containing separate binary vectors or cultures possessing single vectors harbouring multiple expression cassettes.

At the time agroinfiltration was developed, the building blocks of plant-based expression cassettes used for stable transformation were regularly drawn from plant pathogens in recognition of their ability to subvert host controls over gene expression (Fig. 1a). Such elements included the *Cauliflower mosaic virus* (CaMV) 35S promoter [7] and translational enhancer elements from *Tobacco mosaic virus* (TMV) [8] and *Tobacco etch virus* (TEV) [9]. This trend continued through the development of replicating viral vectors and the development of high yielding non-replicating expression systems (for a recent review of the development of such systems, see [10]). Deconstructed viral vectors based on TMV [11], *Cowpea mosaic virus* (CPMV) [12] and *Bean yellow dwarf virus* (BeYDV) [13] have been developed as high yielding expression systems through a reduction to components essential for replication and translation. In the case of the naturally bipartite CPMV, the gene of interest was separated from the *trans*-acting replication functions, permitting the efficient co-expression of multiple proteins [14].

The deployment of suppressors of gene silencing, such as the popular P19 from *Tomato bushy stunt virus* (TBSV), to reduce post-transcriptional gene silencing [15] has had an enormous impact on yields that can be obtained via transient protein expression. Stabilising mRNA in this way enabled the engineering the 5′UTR of CPMV RNA-2 such that, while they would not be replication-competent [16], the synthetic mRNAs bestowed extremely high levels of translation (CPMV-*HT*) [17]. This advance, along with the creation of the bespoke pEAQ vector series specifically designed for transient expression with CPMV-*HT* [18,19], simultaneously removed restrictions on insert length imposed by the need for replication and allowed the efficient high level co-expression of multiple genes (Fig. 1b). This last feature is becoming particularly important considering the ever-increasing complexity of synthetic biology applications.

## Macromolecular complexes

Macromolecular complexes can consist of multiple copies of a single or several polypeptides; in the latter case, the polypeptides can be present in differing ratios. A particular type of complex, virus-like particles (VLPs), has received much attention due to their potential use in nanotechnology and as novel vaccines. Plant virus particles produced via infection, particularly those of CPMV, have attracted considerable interest in this regard [20] but suffer from the disadvantage that they contain infectious nucleic acid. Thus attention has turned to use of synthetic biology approaches to produce nucleic acid-free VLPs.

**Figure 2:**
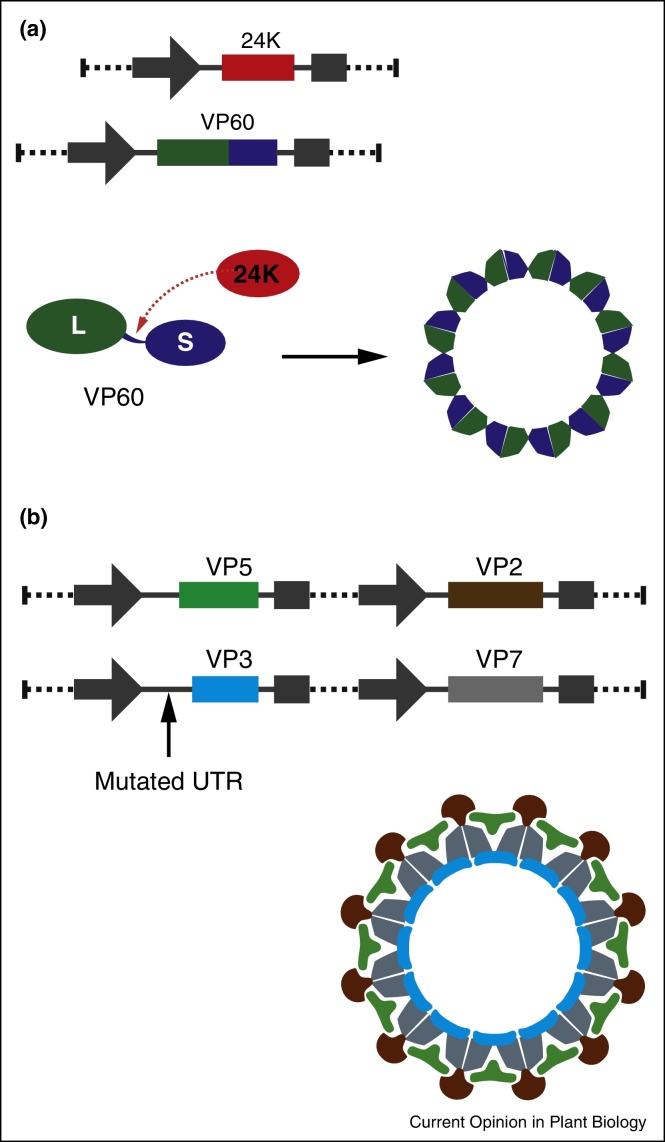
Transient production of VLPs from pEAQ vectors. **(a)** Co-expression of the coat protein precursor (VP60) and the 24K proteinase from CPMV results in assembly of empty VLPs from the large (L) and small (S) coat protein following 24K-mediated VP60 cleavage. **(b)** Co-expression of the four structural proteins of BTV results in the efficient assembly of full VLPs when VP3 expression is restricted through the engineering of the sequence encoding the 5′UTR.

Co-expression in *N. benthamiana* of the CPMV coat protein precursor along with the 24K proteinase using pEAQ vectors (Fig. 2a) results in the efficient assembly and accumulation of empty virus-like particles (eVLPs) comprised of 60 copies each of the processed large and small coat proteins [21]. The absence of RNA, combined with the removal of limitations on coat protein inserts previously imposed by compatibility with virus replication and movement, breathed new life into the use of CPMV capsids as platform in biotechnology and nanotechnology [22,23]. Furthermore, the interior space is now available for encapsulation of various payloads. In an early example, metal ions were reduced following diffusion into the interior cavity resulting in the spatially confined deposition of metals [24]. More recently, the interior surface of eVLPs was specifically functionalised with a variety of molecules including fluorescent dyes, leading to the proof of concept demonstration of tumour targeting with eVLPs [25^•^]. As well as providing material for bionanotechnology, the synthetic reconstruction of CPMV eVLPs also revealed a hitherto unsuspected role of the small coat protein C-terminus in virus assembly [26].

Although the CPMV-*HT* system has been used to generate VLPs from a number of other viruses, such as Hepatitis B core particles [17] and Human [27] and Bovine [28] Papillomavirus particles, all of which consist of a single type of subunit, the system was recently used to reconstruct fully assembled and immunogenic *Bluetongue virus* (BTV) VLPs [29^••^]. This required not only the simultaneous expression of four structural proteins but was optimised by varying the expression levels between the proteins to favour the accumulation of fully assembled particles over assembly intermediates (Fig. 2b). This fine tuning was achieved by engineering the CPMV-*HT* system to reduce overexpression of BTV VP3 relative to the other coat proteins. This resulted in reduced accumulation of assembly intermediates with no impact on the yield of fully assembled VLPs [29^••^]. Expression cassettes derived from CPMV-*HT* have now been developed for modulating expression across a range of levels (unpublished data) that will enable further fine tuning for the construction of complex macromolecular assemblies as well as for the synthetic reconstruction of metabolic pathways as discussed below.

## Engineering metabolism

Although virus-derived transient expression systems have been extensively used for the production of pharmaceutical proteins, including VLPs, in plants, it is only recently that this technology has been used to express active enzymes, with a view to manipulate plant metabolism. This may relate to the fact that the co-expression of the multiple proteins that would be needed to analyse or recreate metabolic pathways is very difficult, if not impossible, to achieve with replicating virus systems due to such phenomena as recombination between different virus constructs and virus exclusion, whereby one construct comes to dominate within a given cell. As a result, replicating systems have been used for the production of single enzymes intended for subsequent purification [30] or for the expression of a transcription factor that can regulate metabolism [31]. By contrast, non-replicating systems such as those based on CPMV-*HT*, do not suffer from these disadvantages and are being increasingly used for synthetic biology applications related to metabolic engineering. The pEAQ vectors, which permit the expression of multiple proteins from the same construct [18,19,32^•^] as well as providing a method the production of individual enzymes for subsequent purification [33,34], have proved extremely useful in this regard.

### Manipulating the synthesis of natural products

Triterpenes are one of the largest classes of plant-derived natural products. They have a wide range of potential commercial applications in, for example, the pharmaceutical, food, and cosmetic industries. However, to date, exploitation of triterpenes has been limited by their recalcitrance to synthetic chemistry and their occurrence in low abundance in complex mixtures in plants. Thus there is a real opportunity to use synthetic biology approaches to both understand and modify the pathways of their biosynthesis.

**Figure 3:**
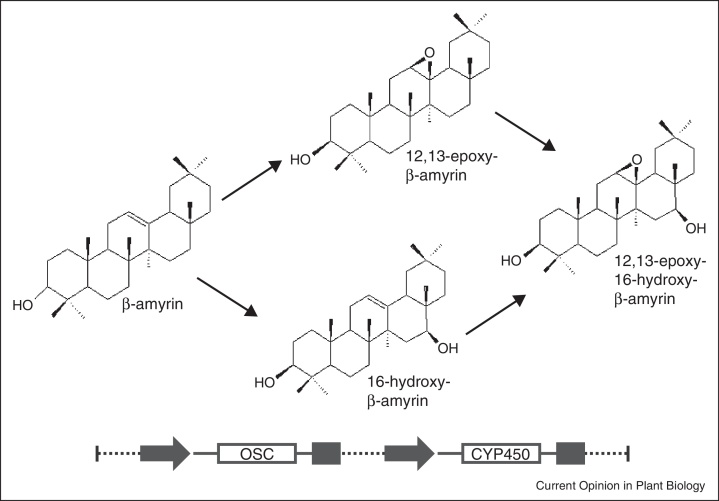
Conversion of β-amyrin to 12,13β-epoxy-16β-hydroxy-β-amyrin by transient co-expression of the first two enzymes of the avenicin biosynthetic pathway, oxidosqualene cyclase (OSC) and a cytochrome P450 (CYP450). Potential intermediates in the synthesis are shown. From [37^••^].

Avenacins are antimicrobial triterpene glycosides that are produced in the roots of oats (*Avena* spp.) to protect the plants against soil-borne pathogens. They possess some unusual structural features not found in other plant triterpene glycosides. For example, they are acylated at the carbon-21 (C-21) position with either N-methylanthranilate or benzoate, and this acylation is important in determining their biological activity. The first example of the use of transient expression to probe avenicin metabolism was the expression of a serine carboxypeptidase-like (SCPL) acyltransferase from oat in *N. benthamiana* using a CPMV-based vector [35]. It was demonstrated that the expressed oat enzyme, SCPL-1, was functional in *N. benthamiana* extracts and was able to catalyze the synthesis of both N-methyl anthraniloyl-derivatized and benzoyl-derivatized forms of avenacin, *in vitro*. Mugford *et al.* [36^•^] subsequently used the same approach to investigate the roles of anthranilate N-methyltransferase (MT1), UGT74H5 glucosyltransferase and SCPL-1 in the synthesis and transfer of the N-methyl anthraniloyl acyl group onto the triterpene backbone. Expression of MT1 alone was sufficient to cause *N. benthamiana* leaves to fluoresce blue under UV illumination and this fluorescence was enhanced when MT1 and UGT74H5 were expressed in combination. Analysis of leaf extracts revealed that coexpression of MT1 and UGT74H5 resulted in the accumulation of N-methyl anthraniloyl-O-Glc. Expression of SCPL-1 acyltransferase together with MT1 and UGT74H5 did not lead to accumulation of any additional fluorescent compounds, indicating that SCPL1 does not acylate endogenous compounds in *N. benthamiana*. The use of the pEAQ vector system allowed further probing of the initial stages of avenicin biosynthesis. In these experiments an oxidosqualene cyclase (OSC) and a CYP450 from oat were expressed in *N. benthamiana* leaves, either separately or in combination. Expression of the OSC alone resulted in the leaves producing β-amyrin, a metabolite not normally produced by this species. Infiltration with a pEAQexpress-based construct containing the sequence of both the OSC and the CYP450 resulted in reduction of the signal for β-amyrin and the appearance of a new peak representing a novel compound [32^•^], which was subsequently shown to be 12,13β-epoxy-16β-hydroxy-β-amyrin, a previously unknown compound (Figure 3) [37^••^]. It was further shown that the C12,13 epoxy group in this new compound was crucial for the antifungal activity of avenicins, a discovery that has implications for triterpene metabolic engineering for synthetic biology applications [37^••^].

As well as the work on triterpenes, the coding sequences for two sesquiterpene synthases from *Artemisia annua*, amorpha-3,11-diene synthase (ADS) and epi-cedrol synthase (ECS), were cloned into pEAQ and expressed in *N. benthamiana* [38]. These two enzymes converted the product farnesyl diphosphate into, respectively, amorpha-4,11-diene and epi-cedrol, which are intermediates in the biosynthetic pathway leading to the production of artemisinin, an important antimalarial drug.

### Relocation of metabolic pathways

A recent study has demonstrated that it is possible to use the pEAQ vector series to transfer pathways for the synthesis of structurally complex natural products to the chloroplast, thereby utilising the reducing power generated by photosynthesis as the primary electron donor [39^••^]. In this study the sequences encoding the three enzymes required for the biosynthesis of dhurrin, (an aromatic defence compound), which are normally located in the endoplasmic reticulum, were fused to the sequence encoding the N-terminal 52 amino acid transit peptide of *Arabidopsis* ferrodoxin (Fd) which has known chloroplast targeting properties. Expression of the modified sequences, either individually or in combination, in *N. benthamiana* leaves, resulted in the enzymes being directed to the chloroplasts. They proved to be active when inserted into thylakoid membranes and to catalyze dhurrin synthesis in a light-dependent manner. This study demonstrated that light-driven synthesis of natural products is possible by relocating the enzymes for their biosynthesis to the chloroplast and represents a novel way of exploiting photosynthesis in synthetic biology.

## Conclusions

A number of trends are emerging in response to the demands of the increasingly complex applications required in synthetic biology. Controlled modulation of transient expression levels will play a big role in plant-based synthetic biology as we expand the toolkit available to recreate metabolic pathways or synthesise multi-component assemblies. Other interesting developments facilitated by transient expression relate to the modification of host post-translational processes by co-expression of accessory proteins. This has been used to change the properties of, or improve the yield and quality of, recombinant proteins. For example, glycosylation pathways have been redirected to yield recombinant antibodies with a human-like glycan profile [40] and endogenous proteolysis has been reduced through co-expression of protease inhibitors to improve yield and quality of recombinant antibodies [41,42]. These studies suggest that such an approach could be used to modulate the activity of endogenous enzymes as a complement to introducing exogenous enzymes in metabolic engineering. These reports have also raised interesting questions relating to the impact of transient expression on the host and the possibility of mitigating this impact. Improved knowledge of the plant response to agroinfiltration will improve understanding of the resources available for transient protein expression, pool of available metabolites, and impact on post-translational processes. Although synthetic biology via transient expression in plants will continue to be informed by fundamental biological research, it will also continue to provide opportunities to unravel biological processes by allowing their reconstruction in heterologous systems.

## References and recommended reading

Papers of particular interest, published within the period of review, have been highlighted as:

• of special interest
•• of outstanding interest
